# The Formation and Retrieval of Holistic Event Memories Across Development

**DOI:** 10.5334/joc.149

**Published:** 2021-02-12

**Authors:** Emma James, Gabrielle Ong, Lisa M. Henderson, Aidan J. Horner

**Affiliations:** 1 Department of Psychology, University of York, York, UK; 2 York Biomedical Research Institute, University of York, York, UK

**Keywords:** Memory, Development of cognition, Autobiographical memory

## Abstract

Event memories consist of associations between their constituent elements, leading to their holistic retrieval via the process of pattern completion. This holistic retrieval can occur, under specific conditions, when each within-event association is encoded in a separate temporal context: adults are able to integrate the information into a single coherent representation. In this study, we sought to replicate the holistic retrieval of simultaneously encoded event elements in children, and examine whether children can similarly integrate across separated encoding contexts. Children (aged 6–7 years; 9–10 years) and adults encoded two series of three-element “events” consisting of an animal, object, and location. In the simultaneous condition, they encountered all three event elements at once; in the separated condition, they encountered each pairwise association separately (animal-object, animal-location, object-location). After encoding, they were tested on the retrieval of each within-event association using a 4-alternative-forced-choice task. We inferred the presence of holistic retrieval using a measure of retrieval dependency—the statistical dependency between retrieval of within-event associations. Memory for the pairs improved across ages, but there were no developmental differences in retrieval dependency. In the simultaneous encoding condition, all three age groups showed retrieval dependency. However, counter to previous studies, retrieval dependency was not observed in any age group following separated encoding. The results from the simultaneous encoding condition support the idea that pattern completion processes are developed by early childhood. The absence of retrieval dependency in adults following separated encoding prevent conclusions regarding the developmental trajectory of mnemonic integration.

An event memory consists of many types of information, yet its retrieval tends to be holistic in nature (Horner & Burgess, 2013; Tulving, 2002). This holistic retrieval is proposed to result from pattern completion processes (Marr, 1971; McClelland et al., 1995), allowing all associated elements to be activated upon presentation of a partial cue. In this study, we examined how integrative encoding and holistic retrieval processes change across development.

If pattern completion processes activate all associated event elements, then it follows that memory for within-event elements should be statistically related to one another: we should be more likely to recall a within-event association if we have successfully recalled an association from the same event. This prediction was examined by Horner and Burgess (2013), who asked participants to visualise person-object-location scenarios at encoding. When tested on each of the constituent associations (person-object, person-location, object-location), participants showed significant interdependence in retrieving pairs from the same events. A similar study suggests that this pattern completion process is already in place for children aged 4 and 6 years (Ngo et al., 2019), although with some evidence that holistic retrieval had not quite reached adult levels. On this basis, the present study first aimed to replicate findings of retrieval dependency in 6-year-olds, and test for further developmental differences in a slightly older group of children (9- to 10-year-olds) and adults. We expected to find evidence of retrieval dependency in all three age groups, but that we might see age-related increases in dependency within our larger sample.

Horner and Burgess (2014) observed similar within-event dependency following the separated encoding of each pairwise association, providing that all possible associations were presented. This finding highlights the capacity of the adult memory system to integrate information across different temporal contexts (Schlichting & Preston, 2015). This integration has been related to anterior hippocampal activity upon presentation of the third pair, which is predictive of subsequent memory (Horner et al., 2015). The resulting memory structures appear less prone to decay than only partially overlapping associations (Joensen et al., 2020).

However, we do not know whether children can similarly integrate associations across separately encoded trials. Related studies suggest that this aspect of memory formation may have a slower developmental trajectory. For example, Schlichting et al. (2016) found that children’s ability to draw inferences across overlapping pairs (i.e., encode AB–AC, infer BC) continued to improve into adolescence, and was associated with the protracted development of anterior hippocampal regions. Relatedly, although 9- to 10-year-olds could make inferences across overlapping pairs in a study by Shing et al. (2019), slower response times indicated that they might achieve this via different mechanisms to adolescents and adults. We predicted that ongoing changes in hippocampal maturation would also affect children’s ability to integrate the third pair into a coherent memory representation if presented. Thus, the second aim of this study was to test whether children and adults differ in their ability to form holistic event memories from separately encoded associations, as measured by retrieval dependency (as opposed to associative inference, cf. Schlichting et al., 2016). Although we might anticipate further development between 9- to 10-year-olds and adults, this study provided an initial test of a dissociation in retrieval processes following separated and simultaneous encoding in children, despite no differences being previously observed in adults (Horner & Burgess, 2014).

## Hypotheses

We pre-registered three hypotheses as follows (*https://osf.io/br23e*):

1. We tested whether holistic retrieval continues to develop across childhood. If such an effect is present, we predicted that 9- to 10-year-olds would show greater retrieval dependency than 6- to 7-year-olds.
2. We tested whether holistic retrieval continues to develop beyond the primary school years and into adulthood. If such an effect is present, we predicted that adults would show greater retrieval dependency than children.
3. We tested whether children are less likely to automatically bind associated event elements than adults. We predicted that event elements presented simultaneously would show more dependency than event elements encoded separately as overlapping pairs; and that this difference would be greater in children than adults (who have previously shown no differences in dependency between simultaneous and separate event encoding conditions).

## Methods

### Participants

The study was approved by the Psychology Research Ethics Committee at the University of York. To ensure appropriate statistical power, we conducted simulations using Ngo et al.’s (2019) retrieval dependency data from 6-year-olds (our youngest age group; simultaneous encoding). With 15 “events” from their narrative condition (Experiment 1), a sample size of 45 provided over 90% power to detect significant differences in dependency between the data and the independent model at the *p* < .001 level. Using data from the “hard” condition—without a narrative (Experiment 2)—we observed that a sample size of 45 provided at least 80% power to detect dependency at *p* < .001. We recruited three Year 2 (average 19 pupils per class) and two Year 5 classes (26 per class) from a North Yorkshire (UK) school to meet this minimum sample size, thereby effectively guarding against participant drop-out and increasing the power to detect a—likely smaller—effect of dependency in the separated condition. A comparable sample of 45 was recruited for the adult group.

The school headteacher provided consent for the classes to take part, and parents and children had the opportunity to opt out (*n* = 1). This resulted in 57 children aged 6-to-7 years (*M* = 7 years; 03 months; 26 male) and 51 children aged 9-to-10 years (*M* = 10;04; 32 male). Seven children contributed only partial data due to absence or technical problems. The matrix reasoning subtest of the Wechsler Abbreviated Scale of Intelligence Second Edition (Wechsler, 2011) was administered to document age group differences in nonverbal ability. Both groups had slightly below-average standardised *t*-scores (6–7 years: *M* = 47.82, *SD* = 7.71; 9–10 years: *M* = 45.47, *SD* = 11.34; these data were not analysed further but are available online). Children received stickers, a certificate, and stationery for taking part.

The adult sample consisted of 45 18- to 35-year-old fluent English speakers recruited from the York Psychology Participant Pool (Mean age 21;04; 3 male). They had slightly above average range nonverbal ability (*M* = 52.76, *SD* = 9.71). An additional ten participants completed the study but were excluded due to ceiling levels of performance (≥95%). The session lasted approximately 30 minutes, and participants received either course credit or £5 payment. Note that this adult sample has previously been published as part of an exploratory analysis (James et al., 2020; Experiment 1).

### Stimuli

We developed two lists of 15 “events” that comprised an animal, object, and location. For each element type, the two lists were matched on age of acquisition rating (Kuperman et al., 2012), concreteness (Brysbaert et al., 2014), and number of syllables. Items of each element type were pseudorandomly combined to create fixed events, avoiding strong semantic associations (e.g., *library-book*). Cartoon illustrations of each item were sourced using a web image search. Spoken word presentations of each item were recorded by a female native English speaker.

### Design and Procedure

Participants completed two *Memory Detectives* sessions, during which they were asked to help *Agent Arnie* to remember things he sees on his adventures. Each session incorporated the encoding and test for either the simultaneous or separated encoding condition, the order of which was counterbalanced across participants. For children, the two sessions were administered on separate days. For adults, both sessions were completed in a single sitting, one immediately after the other. All tasks were programmed in OpenSesame (Mathôt et al., 2012).

#### Encoding

Participants were presented with two (separated encoding) or three (simultaneous encoding) pictures aligned horizontally across the centre of the screen, and heard each one named aloud through the headphones (1 second audio clip per picture). Although the majority of previous studies using this paradigm have presented the stimuli as written words, this presentation modality was adopted to remove reading ability as a constraint in younger age groups (see Ngo et al., 2019, for similar adaptions for simultaneously encoded events). Participants were instructed to try and remember each set of items for a subsequent test, and that they should visualise the items interacting to help them. To avoid adults performing at ceiling, we varied trial times according to age group. For children, items remained on screen for an additional 5 seconds after they had been named, totalling 7 seconds for separated encoding trials and 8 seconds for simultaneous encoding trials. For adults, an additional 1 second was provided, totalling 3 and 4 seconds for separated and simultaneous encoding trials, respectively. The next set appeared after either 500 ms (adults), or after the experimenter clicked to confirm attention was on task (children). The total encoding time per event was thus longer for separately encoded events (21 s for children, 9 s for adults) than simultaneously encoded events (8 s for children, 4 s for adults), a difference that is characteristic of previous studies that show no difference in retrieval dependency between the two conditions (e.g., Horner et al., 2014; Bisby et al., 2018).

For the simultaneous encoding condition, the events were presented in a randomised order. For the separated encoding condition, one association from each event appeared within each of three blocks, with a short break between each block. The association type was balanced across events, incorporating five of each animal-object, animal-location, and object-location pair types in each block.

#### Retrieval

Each pairwise association was tested in both directions (i.e., cue *animal* retrieve *object*; cue *object* retrieve *animal*), making six retrieval trials per event. Each participant was tested on every association once (Set 1) before completing the second set of tests in the opposite cue-target direction (Set 2). In each set, there were three blocks of 15 trials (one trial per event). Association types were split evenly across blocks, such that each block contained 5 trials each of animal-object, animal-location, and object-location associations. Block order was counterbalanced across participants, and participants were given optional breaks after every other block.

For each trial, the cue picture was presented at the top of the screen, and its spoken name played through headphones. Participants were asked to choose which of four numbered pictures underneath was seen with the cue picture, and select their answer using a key press response. To reduce ceiling level performance, adults were given 3 seconds to respond (with missing data treated as incorrect, *M* = .04, *SD* = .04). No timeout was set for children.

### Analysis

Participants who averaged 95% or above were excluded and replaced (*n* = 10 adults). We pre-registered two sets of dependency analyses: one with all remaining data, and a second that excluded events with encoding trials marked for inattention (children only). This exclusion was to ensure that any developmental differences in dependency in the separated condition could not be accounted for by children not attending to all three encoding trials per event. All data processing and analyses were conducted in R (R Core Team, 2015).

#### Accuracy

The accuracy data was analysed using a mixed effects regression model, incorporating fixed effects for age group (6–7 years; 9–10 years; adults), encoding condition (simultaneous; separated) and their interaction. Each predictor was effect-coded. Two orthogonal contrasts were used to test our hypotheses for age group: children (6–7 years and 9–10 years) versus adults; and younger (6–7 years) versus older (9–10 years) children.

We initially incorporated random intercepts for each participant and each “event” (animal-object-location), and then used likelihood ratio tests to determine whether association type (animal-object, animal-location, object-location) or cue type and retrieval type separately (i.e., animal, object, or location for each) improved model fit. We then tested for the inclusion of random slopes for each type of intercept, retaining only those that improved model fit under a criterion of *p* < .2. The final model included random intercepts and encoding condition slopes for participant, event, and association type, as well as random slopes for the effect of age group for each event.

We obtained *p*-values using Satterthwaite approximations (package *lmerTest*; Kuznetsova et al., 2017) to determine statistically significant effects. We report uncorrected *p*-values; Holm-Bonferroni corrections applied across the five contrasts within the model did not affect which predictors were statistically significant.

#### Dependency

Retrieval dependency in each participant’s data was computed using the joint retrieval accuracy of trials sharing a common cue (e.g., cue *animal*, retrieve *object* or *location*) or common target (e.g., retrieval of *animal* when cued by either *object* or *location*). For example, for trials that cued by location, a contingency table was created that captured the number of events where: (1) both the associated object and animal were retrieved successfully (across separate retrieval trials); (2) object was retrieved successfully but animal was not retrieved; (3) animal was retrieved successfully but object was not; and (4) neither object nor animal were retrieved successfully. The total count across the four cells in the contingency table was 15, given that there were 15 events per condition. This process was repeated using each element type as a cue and as a retrieval target, resulting in six contingency tables per participant. Thus, for cue object trials, retrieval success for locations and animals was assessed, and for cue animal trials, retrieval success for locations and objects was assessed. For target location trials, retrieval success was assessed for location when cued separately by objects and animals (and similar for target object and target animal trials).

The proportion of contingent responses (i.e., correct-correct, incorrect-incorrect) for each of the six contingency tables was then calculated and averaged (mean) across all six tables, providing a measure of joint (successful and unsuccessful) retrieval in the data for an individual participant. However, because this measure is influenced by accuracy (individuals with many correct or incorrect responses will necessarily have a high proportion of joint retrievals), it is compared to an *independent model* that predicts the proportion of joint retrieval for each participant if there was no relationship between the memory pairs. This proportion of joint retrieval was calculated as (P_AB_*P_AC_)+((1-P_AB_)*(1-P_AC_)) for each contingency table – e.g., for the retrieval of the object (B) and location (C) when each cued by the animal (A). As above, the participant’s proportion of joint retrieval in the independent model was formed by averaging across all six tables. The final retrieval dependency score used for analysis was the difference in dependency between the data and the independent model, such that a positive value provides evidence for a statistical association between memory for within-event elements over and above that predicted by the participant’s overall memory accuracy. This measure of retrieval dependency is identical to that used in Joensen et al. (2020).

We used the same fixed effects structure described above to model the dependency data. However, because dependency is computed across all events for each participant, our variance in item-level random effects was captured on a broader scale by including a random intercept for event list. The final model included random intercepts for participant and for event list; no random slopes could be estimated. Again, Holm-Bonferroni corrections did not affect model interpretation.

## Results

### Accuracy

**Figure 1:**
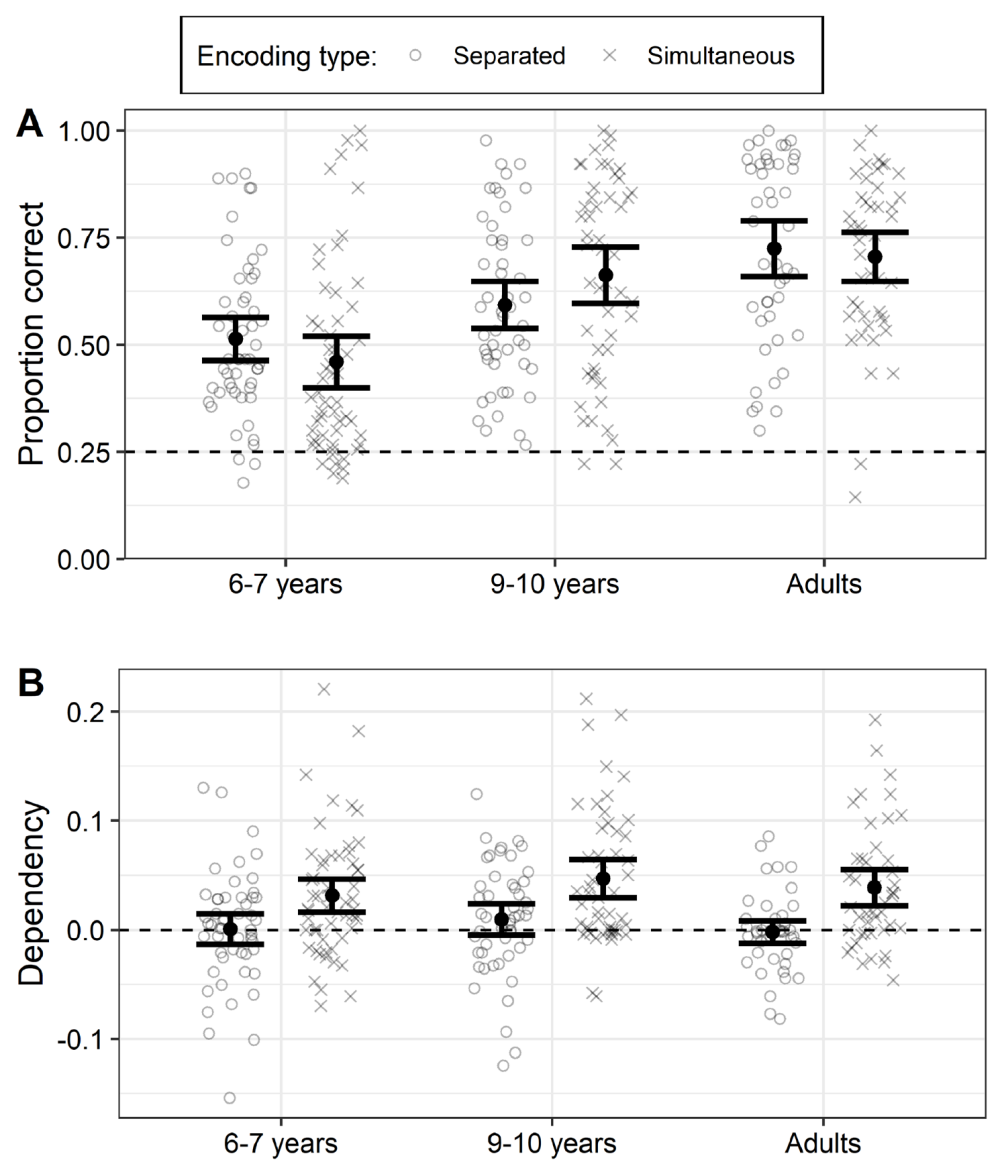
Retrieval accuracy **(A)** and dependency **(B)** following separated and simultaneous encoding of event elements. *Note*: Each data point marks average performance for a single participant, with bold points representing condition means per age group. Error bars mark 95% confidence intervals. In panel A, the dashed line represents chance-level performance. In panel B, the dashed line represents no more dependency in the data than would be predicted by the independent model.

Adults (*M* = .72, *SD* = .18) showed higher accuracy than children (β = 0.28, *SE* = 0.06, *Z* = 4.77, *p* < .001; collapsed across the two younger age groups), and 9-to-10 year-olds (*M* = .63, *SD* = .19) were significantly better than 6-to-7 year-olds olds (*M* = .48, *SD* = .19; β = 0.36, *SE* = 0.10, *Z* = 3.70, *p* < .001). Accuracy did not vary consistently with encoding condition (i.e., separated versus simultaneous encoding) across age groups. The interaction between encoding condition and children vs. adults was not statistically significant (β = –0.06, *SE* = 0.03, *Z* = –1.81, *p* = .070). However, the two child groups showed different patterns of performance across the two encoding conditions (β = 0.14, *SE* = 0.06, *Z* = 2.58, *p* = .010). Post-hoc pairwise comparisons (not pre-registered) showed that 6- to 7-year-old children did not differ in performance across the two encoding conditions (*p* = .427), whereas 9- to 10-year-olds showed higher accuracy in the simultaneous versus separated encoding condition (*p* = .010; see ***Figure 1a***).

### Dependency

#### Full analysis

Retrieval dependency was higher following simultaneous (*M* = 0.04, *SD* = 0.06) than separated encoding (*M* = 0.00, *SD* = 0.05), a difference that was statistically significant (β = 0.02, *SE* < 0.01, *t* = 6.60, *p* < .001). Counter to our hypotheses, there were no differences in dependency across age groups, nor was there any interaction between age group and encoding condition (all *p*s > .12; ***Figure 1b***).

#### Attended-only analysis

Only 0.66% of children’s encoding trials were flagged with inattention. This led to the exclusion of 34 events from analysis, with one child’s separated encoding condition excluded for having <10 remaining events. The pattern of results was identical to the full dependency analysis, with only a main effect of encoding condition (β = 0.02, *SE* < 0.01, *t* = 6.45, *p* < .001).

#### Exploratory analyses

Given the clear difference in dependency between encoding conditions across age groups, we further examined whether there was any evidence for retrieval dependency in the separated encoding condition. We refit the model using cell means parameterisation, providing a test of dependency against 0 for each encoding condition. Dependency was not significantly greater than 0 in the separated encoding condition (β = 0.00, *SE* < 0.00, *t* = 0.61, *p* = .577), but was in the simultaneous condition (β = 0.04, *SE* < 0.01, *t* = 8.02, *p* = .002). This was also the case when analysing the adult sample only: in contrast to previous studies, there was no evidence for retrieval dependency following separated encoding (β = –0.00, *SE* = 0.01, *t* = –0.28, *p* = .780).

## Discussion

Memory for the associations improved across development, with older children remembering more than younger children, and adults outperforming children despite being given shorter trial timings. Despite differences in accuracy, there was no evidence for developmental differences in retrieval dependency. Both children and adults showed statistical dependency between associations encoded at the same time. However, unlike previous studies with adults, we did not observe retrieval dependency across separately encoded associations. We address this discrepancy briefly below.

Our first two hypotheses related to developmental differences in the holistic retrieval of event elements. Ngo et al. (2019) previously found evidence of retrieval dependency in 4-year-olds, but that event-memory coherence was at a lower level than in adults. Six-year-olds did not statistically differ from either 4-year-olds or adults, suggesting an intermediate level of retrieval dependency. Our sample age overlapped with the study by Ngo et al. (2019) to replicate the existence of retrieval dependency in 6-year-old children. Our slightly older group of 9- to 10-year-olds provided an additional test of continued development into adulthood with a larger sample. However, we did not find age-related differences: all three groups showed evidence for dependency in the simultaneous encoding condition. While neuroimaging studies are required to determine whether these behavioural similarities are underpinned by the same neural mechanisms across development, these findings are consistent with the conclusions of Ngo et al. (2019) that pattern completion processes are in place by early childhood.

While we found no developmental differences in holistic retrieval for simultaneously encoded event elements, our third hypothesis predicted that children would be less likely to show retrieval dependency following separated encoding of the within-event associations. Previous studies with adults have found no differences in dependency between simultaneously and separately encoded events (Horner & Burgess, 2014), and dependency following separated encoding is well-replicated (e.g., Bisby et al., 2018; Grande et al., 2019; Joensen et al., 2020). However, here we found that dependency was significantly different between the two conditions across ages, with no evidence for dependency in the separated condition. We subsequently examined this issue in a recent study (James et al., 2020), finding that our inclusion of spoken words had prevented integration across trials in adults. Although the mechanisms for this disruption are currently unclear, it is possible that the presentation of spoken stimuli reduced the time available for visual imagery, encouraged more verbal strategies, or that information presented across multiple modalities better segregated events in memory (resulting in less integration of overlapping material across separate encoding trials). Given this lack of dependency for adults in this study, the current dataset cannot provide a test of developmental differences in integrating across separately encoded trials.

We provide evidence for retrieval dependency (consistent with the process of pattern completion) in two developmental age-groups, replicating and extending a previous study (Ngo et al., 2019). We also assessed whether children could integrate separately encoded overlapping pairwise associations into coherent representations (as indexed by retrieval dependency). Although previous studies have shown evidence for integration in adults, we failed to replicate the effect in an adult sample (see James et al., 2020), not allowing for conclusions to be drawn from the developmental sample.

## Data Accessibility Statement

All experimental materials, data, and analysis scripts are available at *https://osf.io/a8dkj/*.
